# Angiotensin-converting enzyme 2 gene expression in human male urological tissues: implications for pathogenesis and virus transmission pathways

**DOI:** 10.1186/s12301-021-00192-4

**Published:** 2021-07-01

**Authors:** Sammy Al-Benna

**Affiliations:** Department of Surgical Sciences, Faculty of Medicine and Health Sciences, Stellenbosch University and Tygerberg Academic Hospital, Francie van Zijl Drive, PO Box 241, Cape Town, 8000 South Africa

**Keywords:** Urology, Genitalia, Male, Angiotensin-converting enzyme 2 receptor, Coronavirus, Severe acute respiratory syndrome coronavirus 2, COVID-19, Fertility

## Abstract

**Background:**

Severe acute respiratory syndrome coronavirus 2 (SARS-CoV-2) infection occurs through binding and internalization of the viral spike protein to the angiotensin-converting enzyme 2 (ACE2) receptor on the host cell membrane. Pathological changes are caused by damage and failure of vital organs that express high levels of ACE2, including the lungs, the heart and the kidneys. The aim of this study was to investigate ACE2 gene expression in the human male urogenital tract using a public database.

**Methods:**

A search of transcriptomic datasets from a database to investigate ACE2 gene expression in human urogenital tract tissue.

**Results:**

The gene expression profile demonstrated that ACE2 gene expression was higher in human kidney cortex and testis than human lung tissue. The gene expression profile demonstrated that ACE2 gene expression in the human bladder and prostate was comparable to human lung tissue.

**Conclusions:**

Male urogenital tissues are directly susceptible to SARS-CoV-2 infection through the expression of ACE2. Moreover, the SARS-Cov-2/ACE2 interaction may disturb the male genital and reproductive functions.

## Background

Severe acute respiratory syndrome coronavirus 2 (SARS-CoV-2) is an enveloped, positive-sense, single-stranded ribonucleic acid β-coronavirus [1]. SARS-CoV-2 causes the disease called coronavirus disease 2019 (COVID-19) [1–3]. It was first identified in late 2019 in Wuhan, China, and quickly spread to become a worldwide pandemic and public health emergency [1–3]. As of 15 October 2020, a total of 38,789,613 accumulated cases and 1,097,567 deaths are reported worldwide, with an overall mortality rate of about 1% [3]. With this evolving pathology, the fast developing evidence base must be constantly integrated into the present understanding of the disease [4–6].

Epidemiologically, the genome of SARS-CoV-2 consists of 29,891 nucleotides, with an 89% identity to human severe acute respiratory syndrome coronavirus (SARS-CoV) [10, 11]. SARS-CoV-2 infects the human cells by attaching to angiotensin-converting enzyme 2 (ACE2), through its exterior spike (S) protein, modulates the expression of ACE2 and initiates tissue damage [10, 11]. ACE2 is a zinc metalloprotease which shares 42% amino acid homology with angiotensin-converting enzyme (ACE) in its catalytic domain, and consists of 805 amino acids including a C-terminal membrane binding domain and a 17-amino acid N-terminal signal sequence [10, 11]. ACE2 contains one HEXXH zinc-binding motif and is able to hydrolyze angiotensin I (AT1) to produce angiotensin-(1–9) and also has a high affinity for angiotensin II (AT2) to generate angiotensin (1–7) [AT(1–7)] [7]. AT2, the major component of the ACE/AT2/AT1 (AT2 type 1) pathway, facilitates vasoconstriction, promotes cell proliferation and maintains fluid homeostasis [12]. AT(1–7) is an important component of the human renin-angiotensin system (RAS) [12]. It is an endogenous ligand for the G protein-coupled receptor Mas and specifically inhibits AT2 by AT1 receptor antagonism [12]. Furthermore, AT(1–7), the major component of the ACE2/AT(1–7)/Mas pathway is an anti-growth pathway inhibits inflammation, induces vasodilation and is cardioprotective [12].

Tissues that express ACE2 are direct targets of SARS-CoV-2 infection, which can lead to significant pathological changes, tissue organ failure and even mortality in critical cases [14]. Evidence has been accumulating that besides respiratory injury, SARS-CoV-2 also damages other systems, such as the cardiovascular and hepatic [15]. The immune privileged environment of testicles routinely allows immunogenic sperm cells (spermatocytes; spermatids; spermatozoa) to elude the host immune system. However, certain viruses are able to cross between the Sertoli cells of the seminiferous tubules into this immune privileged testicular environment and stimulate an immune response [16]. Systemic viral infections, such as mumps orchitis, can involve the male urogenital system [16]. The seeding and entry of viruses into male reproductive tract cells after SARS-CoV-2 infection is poorly understood and requires further investigation [16]. The aim of this article was to search a gene expression database to find ACE2 expression in tissues of the human urological system.

## Methods

This section presents a search of a public gene expression database (https://www.gtexportal.org/home/) for ACE2 expression in various normal human urogenital tract tissues [17]. The resource database from Genotype-Tissue Expression (Version 8 data release) integrates the expression data of 11,688 normal tissue samples covering 54 tissue types to comprehensively annotate the expression patterns of each gene. Human samples are aligned against the GRCh38 human reference genome. The search provided public RNA sequencing (RNA-seq) data of ACE2 expression. A differential expression analysis was applied on the selected ACE2 dataset to dynamically obtain differentially expressed genes in log_2_(TPM + 1) transformed expression data. The transformed expression data from all tissue samples available were plotted using the box plots available from the GTExPortal website with plots shown as median and 25th and 75th percentiles and dots displayed as outliers if they are above or below 1.5 times the interquartile range. All data are publically available online.

## Results

**Fig. 1:**
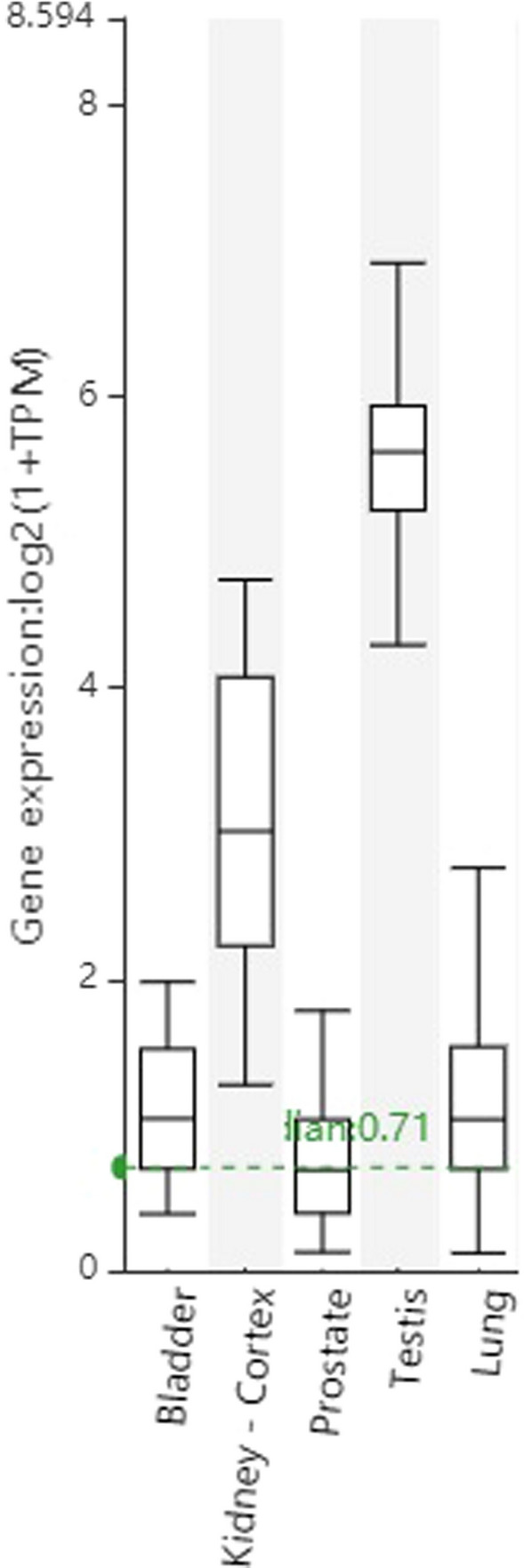
ACE2 gene expression in human urogenital system tissues and human lung tissue

The gene expression database included ACE2 expression profile. The gene expression profile demonstrated that ACE2 gene expression was present in human urogenital tract tissues (Fig. 1). The gene expression profile demonstrated that ACE2 gene expression was higher in human kidney cortex and testis than in human lung tissue (Fig. 1). In addition, the gene expression profile demonstrated that ACE2 gene expression was in the human bladder and prostate was comparable to human lung tissue.

## Discussion

The ACE2 receptor is perceived as the receptor for the passage of SARS-CoV-2 into host cells [10, 11]. SARS-CoV-2 has high proclivity for the ACE2 receptor [10, 11]. SARS-CoV-2 infection is vigorous in cells expressing ACE2 receptor, a type I integral membrane protein that controls cardiovascular and renal functions by negatively modulating the RAS [10, 11]. During virus ingress, the coronavirus S protein requires priming and cleavage at the S1/S2 site of the protein by transmembrane protease serine 2 [11]. The S1 site contains a receptor-binding domain, which then binds to the ACE2 to facilitate virus entry into the cell [1].

This study establishes that ACE2 gene expression in the male urogenital system is greater than or comparable to human lung tissue, the principal corpus tissue whose cells are affected by SARS-CoV-2 infection. Elevated ACE2 expression amplifies virus infection in COVID-19 patients and has been shown to play a role in COVID-19 morbidity and mortality trends, but research on other tissues has not been conducted [13, 18, 19]. Moreover, ACE2 receptor gene expression is upregulated in COVID-19 [18]. ACE2 gene expression level was highest in human male testes, suggesting that they may be more susceptible to the effects of the SARS-CoV-2 virus. The majority of COVID-19 patients present with respiratory symptoms, but little is known about urogenital-related manifestations of the infection [14, 15].

The RAS plays a key role in the modulation of many functions in the body, including the widely known cardiovascular homeostatic mechanisms [12]. The latest developments in tumour pathobiology indicate that local RAS importantly configures the cancer microenvironment and induces angiogenesis, proliferation of tumour cells and metastatic behaviour [20, 21]. Meanwhile, the ACE2/AT(1–7)/Mas axis is associated with anti-proliferative, vasodilatory, antifibrotic and anti-metastatic properties [20]. In addition, the RAS plays a critical role in the testicular function in humans [22, 23]. In Leydig cells, the biological roles of ACE2 involve regulation of production of testosterone and regulation of local vascular systems to balance interstitial fluid volume via modulating conversion of AT2 to AT1 [24–26]. SARS-CoV-2 infection and pathogenesis is predominantly determined by receptor recognition [9]. SARS-CoV-2 has higher binding affinity in cells with higher ACE2 expression, which supports more efficient cell entry [9, 26]. Intervention strategies can only be improved by understanding SARS-CoV-2 cell entry mechanisms.

There are two critical observations with regard to human male urological tissues and the COVID-19 pandemic: (1) adult males have a higher prevalence of severe COVID-19 complications; and (2) children have less severe symptoms with a relative immunity [27, 28]. Previous studies have shown that a variety of viruses may affect men’s testicles such as hepatitis B and C viruses, Epstein–Barr virus, human immunodeficiency virus, human papilloma viruses and mumps virus [23]. Some viruses cause orchitis indirectly through an immune response, while other viruses directly attack the testes [23]. The resultant viral orchitis from either direct or indirect attack can lead to male infertility and testicular tumours [23].

Although SARS-CoV virus has never been detected in SARS patients’ testis tissues, orchitis and spermatogenic cell damage have been demonstrated in these SARS-positive patients [22]. Orchitis with scarce or nil spermatozoa in the seminiferous epithelium, sperm cell destruction, spermatogenic cell apoptosis, thickened basement membrane and leukocyte infiltration were found in all testes acquired at autopsy from individuals who died of SARS-CoV [23].

The Sertoli cells of the seminiferous tubules do not shield against SARS-CoV-2 infection, resulting in compromised gonadal function and aberrant expression of male sex hormones [9]. Significant seminiferous tubular injury, decreased Leydig cells, and mild lymphocytic inflammation have been demonstrated in testes from COVID-19 patients [29]. It is proposed that during peak viraemia, SARS-CoV-2 reaches testicular interstitium, where ACE2 expression is high, via the vascular circulation and Leydig cells may be one of the first targets in the testes, impacting testicular function. Male reproductive systems are susceptible to SARS-CoV-2 infection and significant changes in sex hormones in patients with COVID-19 have been observed, suggesting gonadal function impairment [29, 30]. COVID-19 patients have significantly raised luteinizing hormone serum levels, intimating that SARS-CoV-2 infection may instigate male hypogonadism [30].

Furthermore, when 81 COVID-19-positive male patients were compared to 100 age-matched healthy counterparts, the testosterone-to-luteinizing hormone ratio was dramatically and significantly lower [30]. The serum testosterone-to-luteinizing hormone ratio (as an indicator of male gonadal function) was also suggested as a possible marker of reproductive deficiency caused by SARS-CoV-2 [30]. Changes in the steroidogenic pathways that result from SARS-CoV-2 testicular infection may account for the malfunction of Leydig cells that produce hormone and the lower serum testosterone-to-luteinizing hormone ratio [30]. Furthermore, Sertoli cells in the seminiferous tubules also express ACE2 and may similarly be infected by SARS-CoV-2 [30]. Chemokines and cytokines generated by SARS-CoV-2 infection of Leydig cells and Sertoli cells may attract pro-inflammatory peripheral immune cells such as macrophages, monocytes and virus-specific T cells, which may exacerbate inflammation and orchitis, as recorded by up to one in five COVID-19-positive males crossexamined [30], but could also aid viral elimination. COVID-19-positive males rarely report orchitis; it is hypothesized as the immunosuppressive properties of Sertoli cells of the seminiferous tubules and macrophages in the testes, quash inflammation, and in contrast to other organs constraining virus-related injury of the testes. Nevertheless, COVID-19-associated inflammation may temporarily disturb the cohesion of Sertoli cells of the seminiferous tubules that may impact spermatogenesis [29]. As a result, urogenital symptoms and signs should be assessed and investigated in COVID-19 male patients. Further research using both in vitro and in vivo models is necessary to elucidate the effect of SARS-CoV-2 infection on testes function including hormone manufacture, cohesion and organization of the Sertoli cells of the seminiferous tubules, and germ cell decreases to advance the knowledge of the effect of SARS-CoV-2 and COVID-19 on male reproductive ability.

Two studies have reported the absence of SARS-CoV-2 in the semen of males recovering from COVID-19, while two other studies demonstrated its presence in the semen of active and recovering patients [9, 28, 31, 32]. In contrast to a recent study that demonstrated sparse gene expression of ACE2 in the testis, this study found high ACE2 gene expression in the testis [33].

The ramifications of recoverable SARS-CoV-2 ribonucleic acid in semen stay indeterminate, concerning whether this means animate virus or damaged remaining nucleic acid. Future study, including histopathological diagnosis, tissue culture, electron microscopy, nucleic acid detection and immunofluorescence, can decide whether SARS-CoV-2 can be spread through semen and determine the concerned processes [34]. Post-recovery from COVID-19, recovered male patients who are interested in having children should receive a consultation regarding their fertility to monitor and evaluate their male reproductive function, including sperm analysis and sperm cryopreservation in the early stage of disease, and follow-up of reproductive function in the following months.

## Conclusion

ACE2 gene expression is higher in certain parts of the urogenital system than in the respiratory system, and urogenital system pathologies may ensue. The testes, bladder, and prostate, as well as the respiratory system, may be impacted by SARS-CoV-2 infection, both directly and indirectly via the ACE2 receptor. Adult Leydig cells selectively express ACE2 and play a crucial role in steroidogenesis regulation. ACE2 expression variations between males and females can influence not only potential disease sites and viral reservoirs, but also increased prevalence of severe COVID-19 disease in males. More research into ACE2 gene expression and modulation in the male urogenital tract, as well as other related cell types that may influence ACE2 and SARS-CoV-2 pathogenesis, is needed.

## Data Availability

All data are publically available.

## Abbreviations

ACE: Angiotensin-converting enzyme
ACE2: Angiotensin-converting enzyme 2
AT1: Angiotensin I
AT2: Angiotensin II
AT(1–7): Angiotensin (1–7)
S: Spike
